# Serum selenium levels and subacute thyroiditis: associations with disease course and long-term outcomes in a case-control study

**DOI:** 10.1186/s12902-025-01903-6

**Published:** 2025-03-18

**Authors:** Davut Sakız, Murat Çalapkulu, Muhammed Erkam Sencar, İlknur Öztürk Ünsal, Sema Hepşen, Hayri Bostan, Bekir Uçan, Erman Çakal

**Affiliations:** 1 Faculty of Medicine, Department of Endocrinology and Metabolism, Mardin Artuklu University, Mardin, 47100 Türkiye Turkey; 2 Ankara Etlik Integrated Health Campus, Department of Endocrinology and Metabolism, Ankara, Turkey; 3 Department of Endocrinology and Metabolism, Medicana International Ankara Hospital, Ankara, Turkey

**Keywords:** Selenium, Thyrotoxicosis, Inflammation, Trace element, Granulomatous thyroiditis

## Abstract

**Background:**

Subacute thyroiditis (SAT) is an inflammatory disease that induces thyrotoxicosis. Selenium is an essential trace element in thyroid physiology, which has anti-inflammatory and antioxidant properties. However, the relationship between serum selenium levels and SAT has not been well studied. The objective of this study was to evaluate serum selenium levels in patients with SAT compared to healthy controls and to investigate potential correlations between selenium status and clinical outcomes, including disease severity, delayed remission, recurrence, and the development of permanent hypothyroidism.

**Methods:**

This case-control study included 59 patients with SAT and 50 healthy control subjects. Serum selenium levels were analysed using inductively coupled plasma mass spectrometry.

**Results:**

The serum selenium levels of patients with SAT were 69.10 (24.60–130.20) µg/L, while those of the control group were 64.20 (39.21–106.80) µg/L (*p* = 0.121). A negative correlation was detected between serum selenium levels and erythrocyte sedimentation rate, C-reactive protein, neutrophil-to-lymphocyte ratio, free thyroxine, and pain severity. Serum selenium levels did not significantly differ in terms of the response to initial treatment, recurrence, and permanent hypothyroidism.

**Conclusion:**

The study results showed no significant difference in serum selenium levels between patients with SAT and the control group. These results suggest that although lower serum selenium levels may be associated with a more severe and painful SAT course, there is no impact on the long-term prognosis.

## Introduction

Subacute thyroiditis (SAT) is an inflammatory disease that can cause thyrotoxicosis. Although viral infections and genetic predispositions are thought to be the main causes of subacute thyroiditis, its etiology remains unclear [1]. The most common symptoms include neck pain, tenderness, palpitations, tremors, fever and weakness. Although disease severity varies among individuals, SAT is generally a self-limiting condition. However, sometimes delayed remission, recurrence, and permanent hypothyroidism can occur during the disease course. Studies have been conducted to evaluate these conditions and its course, but no clear cause has been identified [2, 3].

Selenium is found in higher concentrations in the thyroid gland than in other organs, and blood levels have been shown to correlate with this concentration [4]. It is a cofactor for about 25 selenoproteins, including glutathione peroxidase, iodotyrosine deiodinase, and thioredoxin reductase, and has an important role in thyroid physiology [5]. It is also found in the structures of many selenoproteins that regulate the inflammatory response and have antioxidant activity [6, 7]. Multiple studies have indicated that reduced serum selenium levels are correlated with an increased prevalence and severity of autoimmune thyroid diseases [8–10]. However, studies on serum selenium levels in patients with subacute thyroiditis are limited. The primary objective of this study was to evaluate serum selenium levels in patients with SAT compared to healthy controls and to investigate potential correlations between selenium status and clinical outcomes, including disease severity, delayed remission, recurrence, and the development of permanent hypothyroidism. By addressing these gaps, this study aims to provide new insights into the role of selenium in the clinical course of SAT.

## Materials and methods

### Participants

This case-control study recruited a total of 59 patients diagnosed with SAT and 50 healthy volunteers from the Endocrinology and Metabolism Outpatient Clinic. The patients ranged in age from 20 to 73 years, with a mean age of 40.41 ± 13.14 years in the control group, and from 30 to 78 years, with a mean age of 44.36 ± 11.01 years in the SAT group. SAT was diagnosed based on the 2016 American Thyroid Association guidelines, using medical history, physical examination, laboratory findings and thyroid ultrasonography results [11]. SAT was suspected in patients presenting with anterior neck pain radiating to the jaw and ears, and fever, and those exhibiting tenderness and pain in the thyroid lobe during physical examination. It has long been recognized that no significant differences exist among various pain measurement methods when assessing pain [12]. Given its ease of application, we opted for the numerical rating scale ranging from 0 (no pain) to 10 (worst possible pain).

Elevated erythrocyte sedimentation rate (ESR), C-reactive protein (CRP), leukocytosis, and thyrotoxicosis were accepted as laboratory values consistent with SAT. Focal or diffuse, hypoechoic-heterogeneous areas with reduced vascularity that were painful when probed by ultrasound were considered as indicative of SAT. The control group consisted of individuals who underwent a check-up during the same period and had no pathology detected in their test results. Prior to study inclusion, participants were screened for the presence of chronic diseases, previous thyroid diseases, smoking, and alcohol use. Only individuals without a history of chronic diseases or previous thyroid diseases and who did not smoke or drink alcohol were included in the study. None of the participants were taking selenium-containing supplements. The study was approved by the Local Ethics Committee and conducted in accordance with the Declaration of Helsinki. Informed consent was obtained from all the study participants.

### Follow-Up evaluation

All patients were treated with ibuprofen or methylprednisolone, and propranolol was administered for those exhibiting thyrotoxicosis symptoms as needed. Treatment was determined based on the attending endocrinologist’s clinical experience, with the treatment protocol completed within 6 weeks. The dose titration and duration of treatment could be changed based on the patient’s clinical symptoms. All patients who did not achieve remission or had recurrence with the initial treatment received second-line therapy consisting of a 6-week course of prednosilone. A total of 57 patients with SAT continued hospital visits during routine SAT follow-up protocols. Two patients with SAT who did not come to follow-up visits were excluded from the analyses of remission, recurrence, and permanent hypothyroidism. Clinical remission was defined as the resolution of symptoms and signs. Non-remission was characterized by failure to demonstrate clinical improvement and regression within 6 weeks of treatment. The reappearance of clinical and laboratory findings of SAT in patients who had previously recovered was considered disease recurrence. Permanent hypothyroidism was defined as hypothyroidism persisting for at least 6 months and requiring levothyroxine replacement in patients with improved SAT clinical and laboratory findings, but who still had hypothyroidism. The recurrence and hypothyroidism analyses included patients followed up for at least 6 months after the discontinuation of anti-inflammatory treatment (57 patients with SAT).

### Methods

*Serum Selenium Levels*: Blood samples used to evaluate serum selenium levels were obtained pre-treatment during routine examinations and analyses. Samples were collected between 08:00 a.m. and 10:00 a.m. while participants were in a fasting state. To allow complete coagulation and ensure optimal serum yield, blood samples were left undisturbed for 30–60 min. Subsequently, the samples were centrifuged at 3000 rpm for 15 min at room temperature. The serum was then carefully separated using a plastic pipette and stored at − 80 °C until analysis. The serum selenium levels of all the samples were analyzed using inductively coupled plasma mass spectrometry (ICP-MS), using Thermo ICAP ICP-MS (Illinois, USA). The instrument was fine-tuned according to the manufacturer’s requirements before starting each analytical run. Helium was selected as a reaction gas. The calibration curve was plotted with the multi-element calibration standard with predetermined concentrations of 25, 50, 75, 100 and 125 µg/L. The multi-element calibration standard produced by CPA chem (Bogolimovo, Bulgaria) was used. This standard contained the following elements in an 10% HNO3 matrix: Be (19 mg/L), Cd (10 mg/L), Co (10 mg/L), Mn (10 mg/L), Cr (20 mg/L), Cu (20 mg/L), Ni (20 mg/L), Al (40 mg/L), As (40 mg/L), Ba (40 mg/L), Pb (40 mg/L), V (40 mg/L), B (100 mg/L), Fe (100 mg/L), Se (100 mg/L), Tl (100 mg/L), and Zn (100 mg/L). Correlation coefficients for calibration curves were always > 0.999. The blank reagent consisted of high-purity water and Sigma-Aldrich Sigma mix 1 Standard (Taufkirchen, Germany). Seronorm Trace Elements L1–L2 Serum (Billingstad, Norway) was used for accuracy control in the selenium analysis. The reference range for selenium levels, as specified in the manufacturer’s instructions for the device used, is 63–160 mcg/L.

#### Thyroid function tests and acute phase reactants

Laboratory tests levels were measured at the time of diagnosis and then repeated when the treatment was completed and when relapse occured. Thyroid function tests were repeated every 6 weeks in patients with persistent hypothyroidism, and levothyroxine doses were adjusted based on (TSH) levels. Thyroid function tests and thyroid antibodies were measured using an automated direct chemiluminescent immunoassay (Beckman Coulter, CA, USA). Reference ranges were defined as TSH of 0.38–5.33 mIU/L, fT4 of 0.60–1.25 ng/dl, fT3 of 2.28–4 ng/L, anti-thyroglobulin antibody (anti-TG) of 0–40 IU/mL, anti-thyroid peroxidase antibody (anti-TPO) of 0–35 IU/mL, ESR of 0–20 mL/h, CRP of 0–5 mg/L, leukocyte count of 3570–11,010 103 n/µL, neutrophil count of 1690–7550 n/µL and lymphocyte count of 880–2890 n/µL. The neutrophil/lymphocyte ratio (NLR) was calculated as the ratio between the neutrophil and lymphocyte counts.

#### Ultrasonographic examination

All patients underwent an ultrasonographic examination at the time of diagnosis, after treatment completion and at the time of relapse. Ultrasonographic examination was performed by the authors experienced in ultrasonography using Hitachi HI Vision Prerius (Hitachi, Tokyo, Japan), with a linear 13 MHz probe. The thyroid volume was calculated using the ellipsoid volume formula (ml) (length (cm) × width (cm) × thickness (cm) × π × 4/3).

#### Statistical analysis

Data obtained in the study were analyzed statistically using IBM SPSS Statistics software version 23.0. Categorical data were presented as frequencies and percentages. The normality of data distribution was assessed using the Kolmogorov–Smirnov test. Normally distributed continuous variables were expressed as mean ± standard deviation (SD) values, and non-normally distributed variables as median (range) values. The Independent Samples t-test was used to compare normally distributed continuous variables, and the Mann–Whitney U test was applied to non-normally distributed variables. Associations between categorical variables were assessed using Chi-square analysis and Fisher’s Exact test. Correlations between the variables were evaluated using Spearman’s correlation test. The level of statistical significance was set at *p* < 0.05.

## Results

Evaluations were made of 47 female (79.7%) and 12 male (20.3%) patients with SAT, and 33 female (66%) and 17 male (34%) subjects in the control group (Table 1). The female/male ratio of patients with SAT was approximately 4/1. The mean age did not differ significantly between groups (*p* = 0.102). Serum selenium levels were comparable between the SAT and control groups (*p* = 0.121). However, thyroid volume was significantly higher in patients with SAT (*p* < 0.001). Anti-TPO and anti-Tg positivity rates showed no significant difference between the groups (Table 1). The median pain score of patients with SAT was 7 (5–10).

In the SAT group, correlation analysis showed no significant relationship between serum selenium levels and TSH, fT3, leukocyte count, neutrophil count, lymphocyte count, or thyroid volume (*p* > 0.05). However, serum selenium levels were negatively correlated with ESR, CRP, NLR, fT4, and pain score (Table 2).

**Table 1:** Clinical and biochemical parameters of patients with SAT compared to healthy control subjects

	Control	Subacute thyroiditis	*p*
Gender, female/male: number (percentage)	33 (66%)/17 (34%)	47 (79.7%)/12 (20.3%)	0.108
Age (years)	40.41 ± 13.14	44.36 ± 11.01	0.102
Selenium (µg/L) (63–160 mcg/L)	64.20 (39.21–106.80)	69.10 (24.60–130.20)	0.121
TSH (0.38–5.33 uIU/mL )	1.87 (0.41–5.50)	0.020 (0.003–0.37)	< 0.001
fT4 (0.60–1.25 ng/dl )	0.85 (0.68–1.10)	1.63 (0.69–4.25)	< 0.001
fT3 (2.28–4 pg/ml)	3.00 (2.05–3.71)	4.89 (2.44–13.46)	< 0.001
Anti-TPO positivity (n/%)	5 (10%)	3 (5.1%)	0.446
Anti-Tg positivity (n/%)	6 (12%)	2 (3.4%)	0.139
ESR	7 (1–22)	49 (9–120)	< 0.001
CRP (0–8 mg/L )	1.24 (0.12–22.47)	45.7 (4.68–172)	< 0.001
WBC (3570–11010 n/µL )	7095 ± 1689	8613 ± 2464	< 0.001
Neutrophils (1690–7550 n/µL )	4134 ± 1327	5788 ± 2036	< 0.001
Lymphocytes (880–2890 n/µL)	2338 ± 845	2031 ± 643	0.043
NLR	1.66 (0.73–7.00)	2.94 (0.79–8.36)	< 0.001
Thyroid volume (cm^3^)	12.25 ± 4.13	22.11 ± 10.83	< 0.001
Pain Score (0–10)		7 (5–10)	

SAT: Subacute thyroiditis; fT4: Free thyroxine; fT3: Free triiodothyronine; TSH: Thyroid stimulating hormone; ESR: Erythrocyte sedimentation rate; CRP: C-reactive protein; WBC: White blood cell; Anti-TPO: Anti-thyroid peroxidase antibody; Anti-Tg: Anti-thyroglobulin antibody; NLR: Neutrophil -Lymphocyte Ratio.

Normally distributed continuous variables were expressed as mean ± standard deviation (SD) values, and non-normally distributed variables as median (range) values.

**Table 2:** Correlations of laboratory parameters, pain score and serum selenium levels of patients with SAT

		Sedimentation	CRP	fT4	Pain Score	NLR
Selenium	r value	−0.437	−0.293	−0.284	−0.336	−0.329
	*p* value	0.001	0.024	0.036	0.013	0.013

SAT: Subacute thyroiditis; fT4: Free thyroxine; NLR: Neutrophil -Lymphocyte Ratio; CRP: C-reactive protein;

Recurrence occurred in 7 (12%) patients after 6 weeks of treatment, and 8 (13%) patients did not achieve remission after the initial treatment. Of these 8 patients who did not achieve remission, four had been treated with steroids and four with non-steroidal anti-inflammatory drugs (NSAIDs) as first-line therapy. All the patients with recurrence or no remission with the initial treatment responded to second-line (6-week steroid) treatment and achieved remission. None of these patients developed hypothyroidism during the 6-month follow-up period. The serum selenium levels of patients who responded to the initial treatment and those who did not respond were 68.11 (24.60–130.20) and 69.31 (40.80–75.70) µg/L, respectively (*p* = 0.998). The serum selenium levels of patients who developed recurrence and those who did not were found to be 67.16 (58.69–88.58) and 69.30 (24.60–130.20) µg/L (*p* = 0.863), respectively.

Permanent hypothyroidism developed in 5 of the 57 patients who continued to be followed up for 6 months. All of those patients with permanent hypothyroidism had achieved remission with the initial 6-week treatment and did not experience a recurrence. The serum selenium levels of patients who developed permanent hypothyroidism and those who did not were found to be 68.47 (24.60–78.55) and 69.41 (40.80–130.20) µg/L, respectively (*p* = 0.809).

## Discussion

The aim of this study was to examine the relationship between serum selenium levels and the presence and prognosis of SAT. To the best of our knowledge, only one previous study in literature has evaluated serum selenium levels in 25 patients with SAT [13], and no studies have examined the relationship between serum selenium levels and the SAT course. The results of this study demonstrated that serum selenium levels in patients with SAT did not differ from those of the control group. However, a negative correlation was determined between serum selenium levels and the acute phase reactants of ESR, CRP, and NLR. In addition, serum selenium levels were seen to be negatively correlated with fT4 levels and neck pain scores. No association was found between serum selenium levels and response to SAT treatment, recurrence, or permanent hypothyroidism.

Subacute thyroiditis pathophysiology is predominantly influenced by two key processes: HLA-associated genetic predisposition and oxidative stress accompanied by inflammation following possible viral triggers [1, 14]. Preclinical studies have demonstrated that sodium selenite inhibits HLA-DR expression, thereby preventing apoptosis and the secretion of proinflammatory cytokines, which suggests a significant role in the pathophysiology of SAT [15, 16]. The effects of selenium may be further attributed to its function as a cofactor for numerous antioxidant and anti-inflammatory enzymes, including glutathione peroxidase and thioredoxin reductase [17]. For example, the thioredoxin reductase family regulates the oxidation state of cysteine residues [18]. In addition, the activities of peroxiredoxins and methionine sulfoxide reductases depend on the common substrate thioredoxin, which is mediated by thioredoxin reductase [19, 20]. In contrast, the glutathione peroxidase family catalyses the safe breakdown of excess hydrogen peroxide [21], and protects membrane lipids by reducing phospholipid hydroperoxides. They also participate in arachidonic acid metabolism, cell survival control and redox-regulated signalling [22]. Although all these findings emphasize the relationship between selenium and SAT, the number of clinical studies conducted on this topic remains limited.

A previous study by Moncayo et al. reported lower serum selenium levels in patients with SAT compared to a control group, whereas the current study findings showed no significant difference in selenium concentrations between the patients with SAT and the control group [13]. This discrepancy may be attributable to differences in study populations and methodologies used across the two studies. Specifically, the Moncayo et al. study was conducted on a relatively small sample size of only 25 patients with SAT, which could limit the generalizability of their results. Moreover, the absence of detailed demographic, clinical, and laboratory data in the Moncayo et al. study further restricts interpretation of the results. That investigation was unable to evaluate serum selenium in relation to inflammatory markers, thyroid function tests, and the clinical course of SAT. Given the paucity of data on the potential role of selenium in subacute thyroiditis, the aim of the current study was to comprehensively examine serum selenium concentrations in a larger cohort of patients diagnosed with this condition compared to a well-matched control group without thyroid disease. The findings suggest that lower selenium levels may be associated with a more painful presentation of subacute thyroiditis, but long-term outcomes may not be associated with serum selenium levels in patients with SAT. This is further supported by a recent study, which also found that selenium levels did not significantly influence the recovery of patients with (sub-)clinical hypothyroidism [23].

It is well known that selenium has anti-inflammatory properties and prevents acute and chronic inflammation [24]. It has also been demonstrated that CRP and selenium levels are negatively correlated and CRP levels decrease with selenium supplementation [25]. Selenium levels have also been reported to be decreased at high CRP levels [26]. Consistent with these findings, a negative correlation was determined in the current study between selenium levels and ESR, CRP, NLR, and neck pain. However, based on the results of this study, these correlations are not robust enough to significantly impact long-term outcomes.

Deiodinase isoenzymes are selenoproteins that play an important role in thyroid metabolism [27]. Deiodinase enzymes convert fT4 to either fT3 or rT3 [28]. However, in thyrotoxicosis conditions such as SAT, unlike hyperthyroidism, fT4 levels are increased further [29]. Therefore, in thyrotoxicosis, the fT4/fT3 ratio increases more than in hyperthyroidism. This situation is also used in the differential diagnosis of hyperthyroidism and thyrotoxicosis [11]. In the current study, a negative correlation was observed between serum selenium levels and fT4. This may indicate that at lower serum selenium levels, the severity of thyroiditis is higher, resulting in higher fT4 levels, or it may indicate that a decrease in deiodinase enzyme activity in selenium deficiency leads to higher fT4 levels.

Although measurement methods and their usefulness are controversial, the fact that functional techniques assessing selenoprotein activity and concentration, such as glutathione peroxidase and SELENOP, were not used, can be considered to be a limitation of this study [7, 30]. However, the serum samples were examined using inductively coupled plasma mass spectrometry (ICP-MS), which is the most commonly used method in literature [7, 30]. Another limitation is the lack of pre- and post-treatment selenium measurements, which could have provided deeper insights into the dynamic changes in selenium status during the course of SAT. Additionally, other antioxidant molecules related to selenium were not investigated, which could have further elucidated the role of oxidative stress in SAT. The small number of patients included at the outset, due to financial constraints, and the limited number of patients with complications such as delayed remission, recurrence, and persistent hypothyroidism, also represent limitations of this study. Furthermore, due to the absence of previous large-scale studies that could serve as a reference and the relatively rare occurrence of subacute thyroiditis compared to other thyroid disorders, a power analysis could not be conducted prior to the study. Nevertheless, this study can be considered to be of value as it contributes to the understanding of the potential role of selenium in the disease course of SAT.

## Conclusion

In conclusion, the current study results demonstrated that no difference was seen in serum selenium levels between patients with SAT and the control group. Serum selenium levels were negatively correlated with neck pain score, ESR, CRP, NLR, and fT4 levels. There was also no difference in serum selenium levels between patients who developed delayed remission, recurrence and persistent hypothyroidism and those who did not, suggesting that lower serum selenium levels may be associated with a more severe and painful course of SAT, but do not have an impact on long-term prognosis. While these findings shed light on the potential association between selenium and SAT, the limited clinical evidence underscores the need for further research to clarify the implications for disease progression and management.

## Data Availability

The data that support the findings of this study are available from the corresponding author upon reasonable request.
